# Development of a pronunciation teaching perception scale (PTPS) for preservice English language teachers

**DOI:** 10.3389/fpsyg.2022.969917

**Published:** 2022-08-17

**Authors:** Halil Ercan, Ilkay Gilanlioglu

**Affiliations:** Department of Foreign Language Education, Faculty of Education, Eastern Mediterranean University, Famagusta, Cyprus

**Keywords:** ELT, pronunciation, scale, preservice teachers, perception, language teachers, English language, L2 pronunciation

## Abstract

The aim of this present research was to develop a valid and reliable scale to determine preservice teachers' perceptions regarding pronunciation teaching. The research sample consisted of 174 preservice teachers in their fourth year studying in English Language Teaching (ELT) departments in eight different universities in North Cyprus in 2021–2022 the academic year. The data collected within the scope of this study were analyzed through SPSS (ver.24) and SPSS Amos software (ver.24) programs to create a valid and reliable measurement tool for the ELT field. The conducted Exploratory Factor Analysis revealed that the scale consisted of 5 factors and 19 items. Moreover, the Confirmatory Factor Analysis results confirmed the five-factor structure. All the dimensions and the overall scale proved to be highly reliable and fit to be applied in the identification of ELT preservice teachers' pronunciation teaching perceptions. Finally, it is hoped that this new scale will help researchers to investigate pronunciation teaching perceptions in various contexts more reliably.

## Introduction

Pronunciation teaching has always been a demanding part of teaching EFL or ESL. However, it is a crucial part of any language teaching program that claims to be offering high quality of language education. The difficulty posed by pronunciation stems mainly from little exposure to and interaction with native speakers and the differences between the phonological systems of English and other languages. Many language teachers nowadays may not prefer to teach pronunciation in the classroom due to their preferences, past learning experiences, or language proficiency levels. Yet, language learners often view pronunciation as being a very challenging task (Wacholtz, 2003). Previous studies on this topic have shown that most pronunciation problems are not only because of physical articulatory problems but also due to L2's cognitive causes (Baker and McCarthy, 1981; Jones, 1997; Kendrick, 1997; Fraser and Department of Education, 2001; Ahmadi, 2011).

According to Fraser (2011), the problem behind not being able to produce the correct sounds is not only because they cannot physically produce the sounds, but also because they cannot distinguish between the sounds to be able to organize and manipulate them as required in the L2 sound system. Especially, in adult ESL programs, language teachers have difficulties in meeting pronunciation learning needs since they lack teaching skills, confidence, and knowledge about teaching certain aspects of pronunciation. Hence, these studies have shown that methodology, curriculum, and techniques need to be improved to attain global standards.

Regarding methodology, for instance, it is known that the teaching method affects the way non-native preservice teachers learn the target language pronunciation system during their education. For example, if a student is studying English as a foreign language using the Grammar-Translation method, we cannot expect her/him to pronounce every word correctly since there is no emphasis on pronunciation in classes. Jones (1997) states that language teachers who teach the target language through the Auditory-Linguistic or Direct Method attach special importance to pronunciation. Furthermore, in the Auditory-Linguistic method, “pronunciation is taught from the beginning to get students to distinguish between members of minimal pairs in language laboratories” (Larsen-Freeman, 2000, p. 46). Similarly, according to Larsen-Freeman (2002), students are expected to demonstrate communicative evidence by using the target language in Communicative Language Teaching. They are supposed to speak L2 fluently, however, there is no need for complete accuracy.

Derwing and Munro (2009) claimed that speaking with a non-native accent might have significant psychological, social, and communicational consequences in EFL contexts. This will obviously be affecting preservice and in-service teachers' beliefs about teaching pronunciation in the following years when they are placed in their teacher positions in classes. The relevant literature has focused on different aspects of pronunciation teaching. Some work has been done on computer-assisted pronunciation teaching (e.g., Levis, 2007). Other research has focused on examining learners' attitudes to the target language as an important factor that helps learners acquire the correct articulation of the sounds and feel motivated (e.g., McKenzie, 2008).

As far as teacher education is concerned, it is important to reveal the perceptions of preservice teachers about learning and teaching the phonological aspects of EFL as preservice teachers are those who will be the future language teachers. Therefore, their perceptions need to be examined and reflected on systematically. Concerning the past 20 years, there have been many studies in the field of teaching and learning pronunciation to reveal learners' and teachers' preferences (Brown, 1992; Claire, 1993; Fraser, 2000; Yates, 2003; Ocampo-Rodríguez et al., 2019). However, the most recent research has focused on different areas of L2 pronunciation. In a state-of-the-art article, Demir and Kartal (2022) aimed at mapping and analyzing L2 pronunciation articles published between 1977 and 2020 and indexed in the Social Science Citation Index (SSCI) in the Web of Science (WoS) database. As significant results, they revealed the influential sources, featured documents, and authors in the field. Moreover, they mapped the most cited references, publications, and authors to help researchers visualize the intellectual structure of the field of L2 pronunciation by clustering.

More specifically, Suzukida and Saito (2022) examined 40 extemporaneous speech samples gathered by Japanese learners to reveal segmental and suprasegmental factors. Subsequently, they presented different levels of global L2 pronunciation proficiency. Furthermore, in a study conducted by Tsunemoto et al. (2020) on 77 Japanese preservice teachers revealed their beliefs about pronunciation teaching. Their findings confirmed that preservice teachers could be categorized into two distinct profiles defined by contributions of their experience.

The first profile consisted of those who had the quality of language learning/teaching experience and pronunciation-related instruction. The second group was composed of those whose beliefs about pronunciation teaching shape the teachability of L2 pronunciation and approaches used in class. Besides, teacher candidates with more experience were found to be more skeptical regarding learning and teaching L2 pronunciation compared to those with less experience. In line with these findings, regarding the pronunciation teaching perception of preservice teachers, to the best of our knowledge, no research has developed a pronunciation measurement scale for investigating preservice English language teachers' L2 pronunciation teaching perceptions. Hence, the development of such a scale would be a significant contribution to the existing literature in the field.

## Methodology

### Participants

The sample consisted of 174 preservice teachers from eight different ELT departments in North Cyprus in the 2021–2022 academic year. All the departments and programs were accredited by the Higher Education Council in Turkey. The preservice teachers were fourth year ELT students chosen based on the stratified sampling technique (Sharma, 2017). The total population of fourth year teacher candidates were two hundred and eighty-six (286) in those universities and the number of preservice teachers reached was two hundred and twenty (220). Out of the 220 potential participants, one hundred and seventy-six (176) volunteered to fill in the questionnaire. However, two (2) questionnaires were excluded as they were not fully completed. In the end, one hundred and seventy-four (174) qualified as participants in this study.

### Data collection procedure

The study used a mixed-method design which consisted of a qualitative approach followed by a quantitative approach. In order to collect rich qualitative data to address the research questions more effectively, first, semi-structured interviews with preservice teachers were conducted. The interviewees belonged to eight different universities in Kyrenia, Nicosia, Famagusta, and Morphou, Secondly, the obtained data were subjected to thematic content analyses. The resulting 66 items were transcribed, and were placed under three codes, namely attitude, emotion, and motivation. For the validity check, the items were also sent to three field experts to get feedback. The items were revised based on the feedback and then distributed to a target population of 220 preservice teachers in ELT departments. One hundred and seventy-four (174) of them responded. After the quantitative data collection, the replies were typed in the SPSS (ver.24) software program to check whether the items on the scale worked or not. Finally, an exploratory factor analysis was conducted on the scale.

### Data analysis

First, exploratory factor analysis was applied. The inference method that had principal component analysis was used in this analysis. Next, the rotation method used involved Kaiser normalization and direct Oblimin (methods). The most important reason for using this method was the component correlation matrix.

Items with an anti-image value of <0.50, which were distributed across more than one factor according to the pattern matrix, items with an ensemble inference variance value of <0.40, and factors with a difference of <0.10 were removed. According to these parameters, exploratory factor analysis was repeated 18 times and a five-factor structure consisting of 19 items was obtained. Confirmatory factor analysis was performed with Amos software program to confirm the validity of this construct. In the confirmatory factor analysis, model fit values were tested with the maximum likelihood method, and it was found that the model fit values were met with the parameters in the literature (Yaşlioglu, 2017). Finally, after performing exploratory factor analysis, confirmatory factor analysis and concordance validity, the Cronbach Alpha internal consistency coefficient was calculated to determine the reliability of the measurement tool. It was found that all dimensions and the overall scale were significantly reliable. As a result of the conducted analyses, a measurement tool with significant validity and reliability was obtained.

## Results

The KMO value as displayed in Table 1 was 0.860 and Bartlett's Test of Sphericity was significant (χ^2^ = 1,474.092, *p* < 0.001). According to Kaiser (1974), KMO value of 0.80 and above is considered good. Based on all these values, it is seen that the number of samples collected for the scale met the criteria of factor analysis.

**Table 1:** Kaiser meyer olkin (KMO) and bartlett sphericity tests.

**Kaiser-meyer-olkin measure of sampling adequacy**		**0.860**
	χ^2^	1,474.092
Bartlett's Test of Sphericity	*df*	171
	*p*	0.000

**Figure 1:**
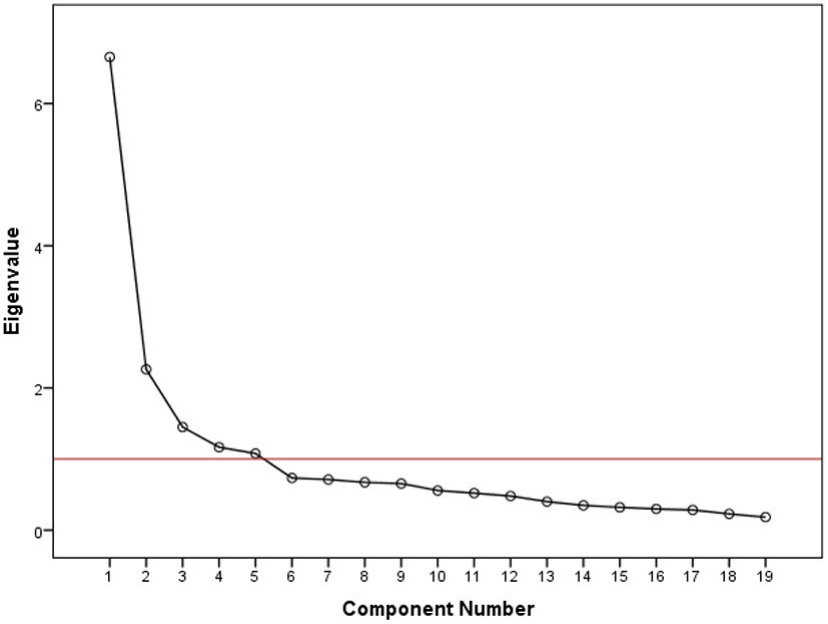
Scree plot chart.

As can clearly be seen in the Scree Plot chart above (Figure 1), the red line represents the point value 1. Furthermore, five different cut-off points on the red line correspond to the five sub-dimensions of the developed scale.

When the values obtained from this scale were examined, it was seen Table 2 that the lowest factor loading value was 0.508 and the highest factor loading value was 0.852. According to Tabachnick and Fidel (2011), the lowest factor loading value was supposed to be 0.32. However, all the values obtained in this study were found higher than the lower limit of 0.32. On the other hand, the total explained variance rate was calculated as 66.36%. The explained variance rate in this study, which is supposed to be at least 50% (Yaşlioglu, 2017), appeared to be well above the lowest limit, i.e., 16.36% greater than the minimum value. Besides, the communalities extraction variance value in this scale, which is supposed to be at least 0.50 (Yaşlioglu, 2017), was found 0.508, which appeared to be slightly above the lowest limit. As for the factors, the first factor consisted of five items and its Eigenvalues value was 6.654. Further, the lowest factor loading value was 0.508 while the highest factor loading value was 0.765. The second factor consisted of two items and its Eigenvalues value was found 2.262. Moreover, the lowest factor loading value was calculated as 0.844 and the highest value was found 0.851. The third factor consisted of 5 items and its Eigenvalues value was calculated as 1.450.

**Table 2:** Distribution of the scale by factors, item factor loads and factor variances.

**New item number**	**Old item number**	**Communalities extraction variance**	**Fac. 1**	**Fac. 2**	**Fac. 3**	**Fac. 4**	**Fac. 5**
1	s62	0.600	0.765				
2	s61	0.678	0.732				
3	s59	0.632	0.689				
4	s60	0.613	0.668				
5	s53	0.518	0.508				
6	s33	0.758		0.851			
4	s34	0.806		0.844			
8	s36	0.730			0.852		
9	s37	0.735			0.765		
10	s41	0.524			0.696		
11	s35	0.600			0.641		
12	s43	0.505			0.530		
13	s46	0.715				0.773	
14	s49	0.668				0.771	
15	s47	0.614				0.720	
16	s56	0.782					−0.836
17	s51	0.752					−0.828
18	s57	0.776					−0.817
19	s54	0.606					−0.665
			**Fac. 1**	**Fac. 2**	**Fac. 3**	**Fac. 4**	**Fac. 5**
Eigenvalues			6.654	2.262	1.450	1.166	1.078
Explained variance value %			35.023	11.903	7.630	6.139	5.672
Total variance %			66.366				

On the other hand, the lowest factor loading value was obtained as 0.530 and the highest value was calculated as 0.852. The fourth factor consisted of three items and its Eigenvalues value was found as 1.166. The lowest factor loading value was found 0.720 and the highest factor loading value was calculated as 0.773. The last factor consisted of four items and its Eigenvalues value was obtained as 1.078. The lowest factor loading value was found 0.665 and the highest factor loading value was calculated as 0.836.

### Confirmatory factor analysis findings

Table 3 below presents the findings of the confirmatory factor analysis.

**Table 3:** Confirmatory factor analysis model fit values.

**Model fit values**	**ELT preservice teachers'pronunciation teaching perception scale values**	**Excellent fit values**	**Acceptable fit values**
χ^2^/*df*	1.477	≤ 3	≤ 4–5
RMSEA	0.053	≤ 0.05	0.06–0.08
NFI	0.865	≥0.95	0.94–0.90
TLI	0.94	>0.95	>0.80
IFI	0.952	≥0.95	0.94–0.90
CFI	0.951	≥0.95	0.90–0.95
GFI	0.897	≥0.95	0.90–0.95
AGFI	0.861	≥0.90	0.85–0.95
RMR	0.06	≤ 0.05	0.05–0.10

Table 3 above displays the acceptable and the excellent fit values and the values obtained in the study. For the excellent and the acceptable fit values, several claims have been put forward in the literature. For instance, the χ^2^/*df* value according to Wheaton et al. (1977) is supposed to be below 5 while for Tabachnick and Fidell (2007) it is supposed to be below 2 to satisfy the fit value condition. The value obtained in this study (χ^2^/*df* = 1.477) met the value of excellent fit. It was stated by Yaşlioglu (2017) that the closer the RMSEA value to 0.1, the worse the fit, and the closer it is to 0, the better the fit.

## Discussion and conclusion

**Figure 2:**
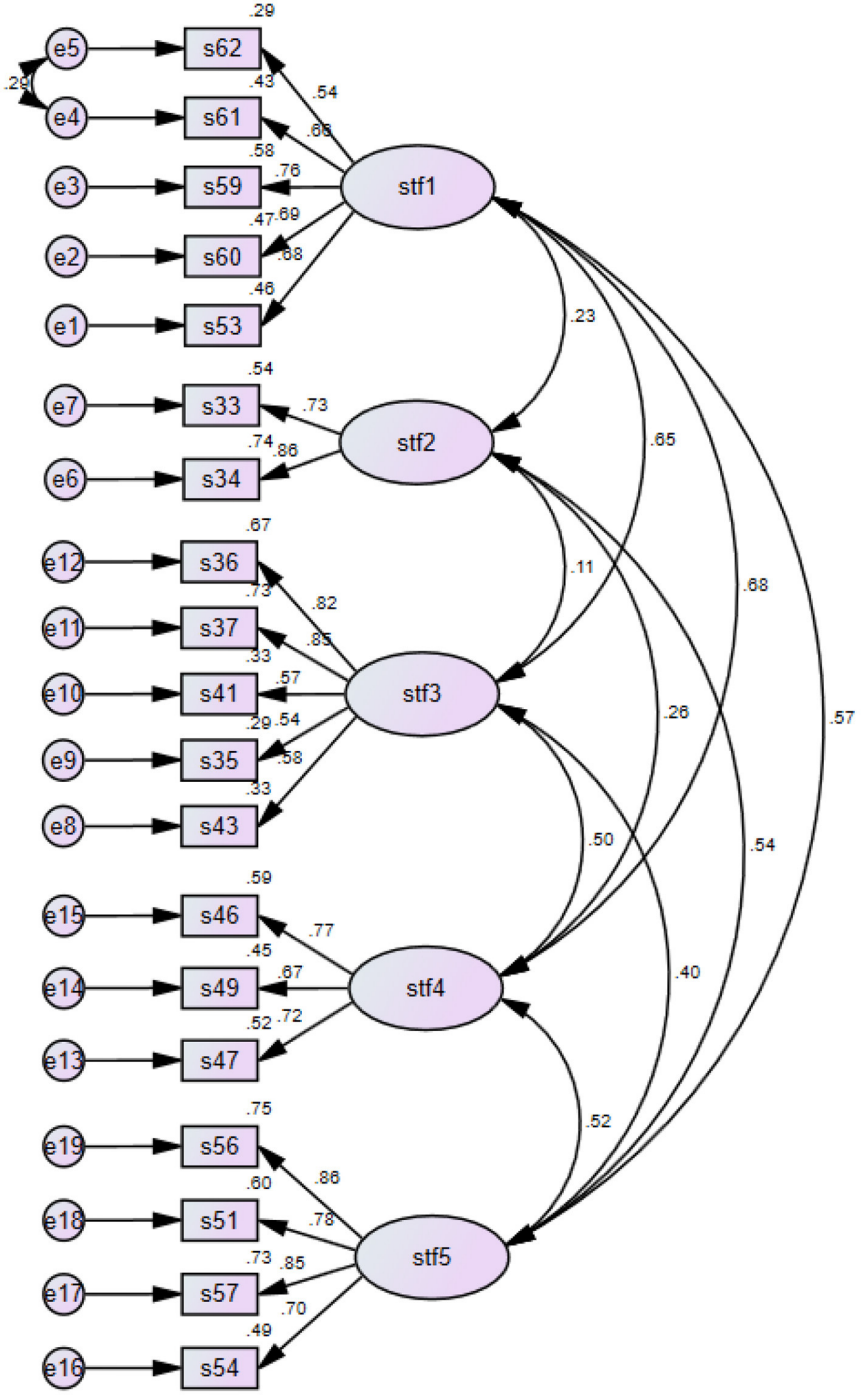
Confirmatory factor analysis factor distribution path diagram.

The RMSEA value in this model, which is among the acceptable reference values, was calculated as 0.53. On the other hand, the NFI value was found 0.865, which is slightly below the acceptable fit value. According to Mulaik et al. (1989), the NFI value is accepted as low in samples smaller than 200. The sample size (n.174) in this study is <200 and it affects the NFI value below 0.90. Since the sample value was low in this study, TLI value was supposed to be checked as stated by Yaşlioglu (2017). According to Byrne (2011), this value (0.940) above the threshold value of >0.80, which is slightly below the 0.95 value, was considered excellent value. Therefore, it can be stated that a good value was achieved based on this model. The IFI value of this scale was calculated as 0.952. It is above the 0.95 value and is considered excellent (Yaşlioglu, 2017). When the CFI value was examined, the value of the model, which had a value above 0.95 and accepted as an excellent fit according to Bentler and Bonett (1980), was found 0.951. The GFI value of this model was calculated as 0.897, which was slightly below the lower limit of 0.90 and this value is sensitive to the sample size. Therefore, it is thought that it affects the fact that it is below 0.90. On the other hand, the AGFI value was found 0.861 and was above the lower limit of 0.85, providing the acceptable fit reference value. As for the RMR value, it was found 0.60, and a value between 0.05 and 0.10 indicates that it meets the acceptable parameters. According to the sources in the literature, it was seen that the model fit values of this study met the acceptable fit condition, and some values met the perfect fit values (Schermelleh-Engel and Moosbrugger, 2003; Tabachnick and Fidell, 2007; Byrne, 2011; Yaşlioglu, 2017). Furthermore, confirmatory factor analysis factor distribution path diagram below (Figure 2), which outlines the correlations between the factors, supports the findings obtained in the study.

Figure 2 above displays the distribution of five factors obtained from the confirmatory factor analysis. It clearly presents the relationship of each factor with another in percentages. For instance, the relationship between the first factor and the fourth factor is 68%.

## Reliability findings

The following table (Table 4) displays the Cronbach-Alpha reliability analysis findings of the scale.

**Table 4:** Cronbach-alpha reliability analysis findings.

**Sub dimensions**	**Number of participants**	**Number of items in sub-dimension**	**Cronbach-alpha internal consistency coefficient (α)**
**Fac. 1**	*174*	5	0.806
	*174*	2	0.768
	*174*	5	0.797
	*174*	3	0.759
	*174*	4	0.871
	*174*	19	0.890

According to Kiliç (2016), a Cronbach-Alpha value above 0.7 is considered good and 0.9 is excellent. All the values in this model were above 0.70 (ranging from 0.759 to 0.890 across the sub-dimensions) and therefore all dimensions and the overall scale appeared highly reliable.

Table 5 below shows the descriptive statistics of preservice teachers' pronunciation teaching perceptions scale items.

**Table 5:** Pronunciation teaching perception scale (PTPS) for preservice English language teachers.

**New item number**	**Old item number**	**Item description**	**x**	**ss**
**Factor 1. Classroom Context**		
1	s62	English pronunciation must be taught in English classes.	4.13	0.937
2	s61	English pronunciation skills are important in English classes.	4.22	0.868
3	s59	English pronunciation can be acquired with practice in class.	4.15	0.874
4	s60	English pronunciation can be acquired with exposure in class.	4.05	0.875
5	s53	I feel better when I overcome a pronunciation problem in class.	4.17	1.000
**Factor 2. Out-of-class Context**		
6	s33	I use English in public (e.g., restaurants, shopping centers).	3.51	1.201
4	s34	I use English outside the classroom.	3.79	1.062
**Factor 3. Learning Styles**		
8	s36	I learn English pronunciation better through explanations in class.	3.71	0.948
9	s37	I learn English pronunciation better through examples in class.	3.93	0.910
10	s41	I learn English pronunciation better through gestures in class.	3.74	1.041
11	s35	I learn English pronunciation better through demonstrations in class.	3.70	0.987
12	s43	I like learning English pronunciation at school.	3.83	1.114
**Factor 4. Beliefs about Learning English**		
13	s46	I like learning English because it is one of the most interesting languages.	4.22	0.911
14	s49	I like learning English as it makes me feel proud.	4.23	0.909
15	s47	I like learning English because it helps me to improve academically.	4.38	0.771
**Factor 5. In-class Activities of Interest**		
16	s56	I enjoy communication in English in class.	4.12	0.920
17	s51	I enjoy speaking English in class.	4.19	0.993
18	s57	I enjoy discussions in English in class.	3.94	1.087
19	s54	I enjoy acting in English in class.	3.84	1.090

A brief discussion of the relevance and potential contribution of our study to the existing literature is in order here. Some studies have focused on examining segmental and suprasegmental factors to distinguish different levels of global second language pronunciation proficiency (Suzukida and Saito, 2022) and some focused on examining the overall effect of mobile devices on L2 pronunciation (Tseng et al., 2022). On the other hand, some have attempted to reveal the pronunciation improvement of certain English consonantal sounds (Ercan and Kunt, 2019). A number of studies have looked into preservice teachers' beliefs and perspectives regarding pronunciation teaching. For instance, a study conducted by Tsunemoto et al. (2020) on 77 Japanese preservice teachers revealed their beliefs about pronunciation teaching. Results indicated that educators should encourage teacher candidates to emphasize on L2 pronunciation teaching. It is considered that our newly developed scale would contribute to such contexts such as the Japanese context to reveal more evidence of the perceptions of preservice teachers and shape targeted dimensions for future teacher candidates.

Parallel to research on perceptions, a study that could be regarded as a close match to ours in terms of instrumentation, investigated ELT department students' deeper understanding of pronunciation problems and pronunciation teaching revealed that it was possible to improve pronunciation skills (Yavuz and Keser, 2019). Since the study adopted and used 12 Likert scale items by Ducate and Lomicka (2009), it could be forecasted that richer data could be gathered through our newly developed scale in such ELT contexts to provide the literature with more specific findings regarding pronunciation teaching perceptions of preservice teachers as it has 5 factors and met excellent fit value (χ^2^/*df* = 1.477).

To sum up, the current study had its own focus on developing an effective tool for identifying preservice teachers' pronunciation teaching perceptions in EFL/ESL contexts around the world. Conducted exploratory and confirmatory factor analyses confirmed that the scale had five factors as *Classroom Context, Out-of-class Context, Learning Styles, Beliefs about Learning English*, and *In-class Activities of Interest*. As the Cronbach-Alpha values in our model were above 0.7 (see Table 4) and the χ^2^/*df* value was 1.477, it could be concluded that the developed scale was a highly reliable tool for revealing preservice teachers' perceptions regarding pronunciation teaching (Tabachnick and Fidell, 2007; Kiliç, 2016; Yaşlioglu, 2017). Consequently, the Pronunciation Teaching Perception Scale (PTPS) designed for the stated purpose proved to be an appropriate and effective instrument (see Appendix 1). Finally, the major implication for the ELT field is that the use of such a scale would help to identify pronunciation teaching perceptions more reliably, and, therefore, provide evidence on which the appropriate teaching techniques, methods, and the curriculum will be based.

## Data availability statement

The original contributions presented in the study are included in the article/supplementary material, further inquiries can be directed to the corresponding author.

## Ethics statement

The current study involving human participants was reviewed and approved by the Scientific Research and Publication Ethics Board of the Eastern Mediterranean University (Reference No: ETK00-2021-0131, Issue: 91). The patients/participants provided their written informed consent to participate in this study. Written informed consent was obtained from the individual(s) for the publication of any potentially identifiable images or data included in this article.

## Author contributions

Both authors listed have made a substantial, direct, and intellectual contribution to the work and approved it for publication.

## Conflict of interest

The authors declare that the research was conducted in the absence of any commercial or financial relationships that could be construed as a potential conflict of interest.

## Publisher's note

All claims expressed in this article are solely those of the authors and do not necessarily represent those of their affiliated organizations, or those of the publisher, the editors and the reviewers. Any product that may be evaluated in this article, or claim that may be made by its manufacturer, is not guaranteed or endorsed by the publisher.

## Appendix

**Appendix 1:** Pronunciation Perception Scale for Preservice English Language Teachers. Instructions This survey is designed to identify preservice English language teachers' pronunciation teaching perceptions. Please read the statements carefully and tick the response (l, 2, 3, 4 or 5) on the answer sheet that expresses your opinion best about each statement. There are no right or wrong answers to these statements. Please make no marks on the items. If you have any questions, please inform the researcher(s) immediately.

	**Pronunciation Teaching Perception Scale (PTPS) for Preservice English Language Teachers**					
**Item No.**		**1: Strongly Disagree** **2: Disagree** **3: Neutral** **4: Agree** **5: Strongly Agree**				
		**1**	**2**	**3**	**4**	**5**
**Factor 1. Classroom Context**						
1	English pronunciation must be taught in English classes.					
2	English pronunciation skills are important in English classes.					
3	English pronunciation can be acquired with practice in class.					
4	English pronunciation can be acquired with exposure in class.					
5	I feel better when I overcome a pronunciation problem in class.					
**Factor 2. Out-of-class Context**						
6	I use English in public (e.g., restaurants, shopping centers).					
7	I use English outside the classroom.					
**Factor 3. Learning Styles**						
8	I learn English pronunciation better through explanations in class.					
9	I learn English pronunciation better through examples in class.					
10	I learn English pronunciation better through gestures in class.					
11	I learn English pronunciation better through demonstrations in class.					
12	I like learning English pronunciation at school.					
**Factor 4. Beliefs about Learning English**						
13	I like learning English because it is one of the most interesting languages.					
14	I like learning English as it makes me feel proud.					
15	I like learning English because it helps me to improve academically.					
**Factor 5. In-class Activities of Interest**						
16	I enjoy communication in English in class.					
17	I enjoy speaking English in class.					
18	I enjoy discussions in English in class.					
19	I enjoy acting in English in class.					
